# Rendering visual events as sounds: Spatial attention capture by auditory augmented reality

**DOI:** 10.1371/journal.pone.0182635

**Published:** 2017-08-08

**Authors:** Scott A. Stone, Matthew S. Tata

**Affiliations:** Department of Neuroscience, Canadian Centre for Behavioural Neuroscience, University of Lethbridge, Alberta, Canada; Centre de neuroscience cognitive, FRANCE

## Abstract

Many salient visual events tend to coincide with auditory events, such as seeing and hearing a car pass by. Information from the visual and auditory senses can be used to create a stable percept of the stimulus. Having access to related coincident visual and auditory information can help for spatial tasks such as localization. However not all visual information has analogous auditory percepts, such as viewing a computer monitor. Here, we describe a system capable of detecting and augmenting visual salient events into localizable auditory events. The system uses a neuromorphic camera (DAVIS 240B) to detect logarithmic changes of brightness intensity in the scene, which can be interpreted as salient visual events. Participants were blindfolded and asked to use the device to detect new objects in the scene, as well as determine direction of motion for a moving visual object. Results suggest the system is robust enough to allow for the simple detection of new salient stimuli, as well accurately encoding direction of visual motion. Future successes are probable as neuromorphic devices are likely to become faster and smaller in the future, making this system much more feasible.

## Introduction

Attentional orienting mechanisms allow us to notice important sensory events and reallocate perceptual resources to deal with them. A variety of visual events are known to trigger reorienting—particularly motion stimuli and abrupt onsets of new objects [1, 2] and the ability to detect these attentional cues is critical to safely interacting with the world. Indeed, visual deficits or impairment of the attention orienting system due to stroke can be debilitating. Fortunately, visual events often coincide with auditory events, thus providing a multimodal cue. For someone with a visual deficit, this coupling of auditory and visual information is critical because it affords the only indication of a potentially important change in the sensory world. However, not all important sensory events are multimodal. For example, the driver of a car might try to alert pedestrians with a horn (purely auditory) or might instead flash the headlights (purely visual). Likewise, objects that start moving in a cluttered auditory scene might not be heard above the background noise floor. Failure to notice such events constitutes an important safety hazard for people with visual impairments. Here we report preliminary success in developing an auditory augmented reality system that renders visually salient events (onsets and motion) onto the spatial auditory scene to provide auditory cues about the visual world.

The perception of auditory motion is largely dependent on the ability to detect the speed and direction of the event. Speed and direction can be perceived through use of the interaural time difference (ITD) and interaural level difference (ILD) cues [3]. It is believed that spectral notches performed by the pinnae can provide an additional cue about the elevation of the sound. The ITD cue is encoded by the difference of arrival times of sound at each ear [4]. That is, a sound source closer to the right ear will enter the right ear up to around 700us before entering the left ear. This small amount of time is dependent on the distance between the ears and the azimuthal arrival angle. The ITD cue can be used by the auditory system to calculate the angle of the sound source. When one ear is closer to the sound source, the sound will have a higher intensity relative to the other ear. This difference in sound intensity is known as ILD. For a review of sound localization techniques, see [5]. When these cues are implemented using software and headphones, the percept of a localizable sound source is apparent. In the same way that someone may localize the auditory motion percept of a car as it passes by, a software-generated sound source can also be panned through virtual space. A key difference is that listening in the free acoustic field (i.e. not through headphones) provides extra cues as to the source elevation and distance. It is worth noting that these cues typically need to be interaural in nature, as monaural cues do not sufficiently code elevation [6]. When using only the ITD and ILD cues through headphones, high-resolution localization is possible, but only in along the azimuthal dimension.

It has been demonstrated that nonindividualized head related transfer functions (HRTFs) can result in accurate localization [7]. Wenzel [7] showed that participants could localize the direction of narrowband noise when using a representative subject’s HRTF. More recent works have focused on measuring individualized head related transfer functions for auditory scene synthesis [8, 9], suggesting that individualized HRTFs maintain the spectral cues important for resolving location. These methods carry the benefit of higher accuracy, but are much more difficult and time-consuming to implement. ITD and ILD cues can be sufficient for azimuthal localization.

An important computational challenge arises when attempting to render visual events into auditory events: a typical frame-based video camera provides highly detailed raw information about color and luminance at frame rates of around 30 frames-per-second. Extracting important events from the dynamics across such frames is computationally intensive, and the Nyquist limit of 15 Hz imposes an upper limit on the temporal resolution. Biologically, conventional frame-based cameras are analogous to the parvocellular and the ventral visual pathway, which conveys high spatial resolution, texture, and colour information, but is slow [10, 11]. By contrast, the visual pathway thought to drive the posterior parietal attention orienting system, the magnocellular dorsal pathway, is fast, low-resolution and insensitive to colour. The ideal camera system for an attentional augmented reality would forgo the high computational demands of a parvocellular-like frame-based camera, and instead emulate the fast, low-resolution dynamics of the magnocellular system. For this reason we designed our augmented reality system not around a frame-based camera, but around a neuromorphic Dynamic Vision Sensor (DVS) [12, 13].

Neuromorphic sensors are based on the design of silicon chips that mimic the underlying function of biological sensory systems (such as the retina and cochlea). A dynamic vision sensor silicon retina approximates the basic information processing pipeline of the human retina: the sensor sends spikes to a computer. The spikes represent log intensity (brightness) changes. The output of the camera signifies the relative changes in scene reflectance. Moving edges and sudden luminance changes are salient to the DVS and generate bursts of spikes, while slow changes and isoluminant edges do not. The neuromorphic retina uses an address-event-representation (AER) system, which allows for the timestamped ordering of temporal contrast events tagged with their spatial coordinates. Due to the way the AER system works, no temporally redundant data is captured; if there are no triggered events on the sensor, no information is sent on to a processing device. By contrast, a conventional frame-based camera sends frames continuously. Since the only information forwarded by the neuromorphic sensor is related to attentionally-relevant events in the scene, it is possible with minimal computation to render visual events into to an augmented auditory space while still maintaining the spatial characteristics of the scene and with a reduced demand on power and computational resources. Early work in auditory augmented reality systems resulted in the SeeHear system: an aid device for the blind [14, 15]. A lens projected light onto a 15x11 matrix of photoreceptors which calculated the light intensity at each point. These intensities were then propagated along a delay line to simulate the time delay of the sound in air. The chip would then output a stereo signal mimicking the spatial properties of the sound in a pair of headphones. Essentially, the device could convert a visual stimulus in motion into an auditory stimulus. The psychophysical efficacy was never evaluated in human studies, so it is unknown how useful such a device would be for users. Other auditory augmentation hardware/software, including the VIS2SOUND [16] and TESSA [17] have also demonstrated that it is possible to convert visual events into spatial auditory events but no human trials have been conducted.

We created an augmented visual-to-auditory system which takes a neuromorphic visual input and augments it to auditory space while still preserving the spatial characteristics of the scene using the ITD and ILD cues. A user of the device experiences visual onsets as bursts of auditory clicks at some azimuthal angle related to the position of the AER visual event. Likewise, a continuously moving visual object is heard as a train of clicks that pans through auditory space with speed and direction related to the visual stimulus. This visual stimulus used was a large white dot on a LCD computer screen either stationary or moving. The algorithm detects the centroid of the stimulus to use as the location of the dot. As a proof-of-concept we show here that a blindfolded listener with almost no training can detect visual onsets and can determine the direction of a visual stimulus in motion at varying speeds and displacements.

## Methods

### Participants

Nineteen individuals from the University of Lethbridge participated in the present study. The study was approved by the University of Lethbridge Human Subject Research Committee (protocol #2013–037), and all participants gave written informed consent prior to participating.

### Apparatus

**Fig 1:**
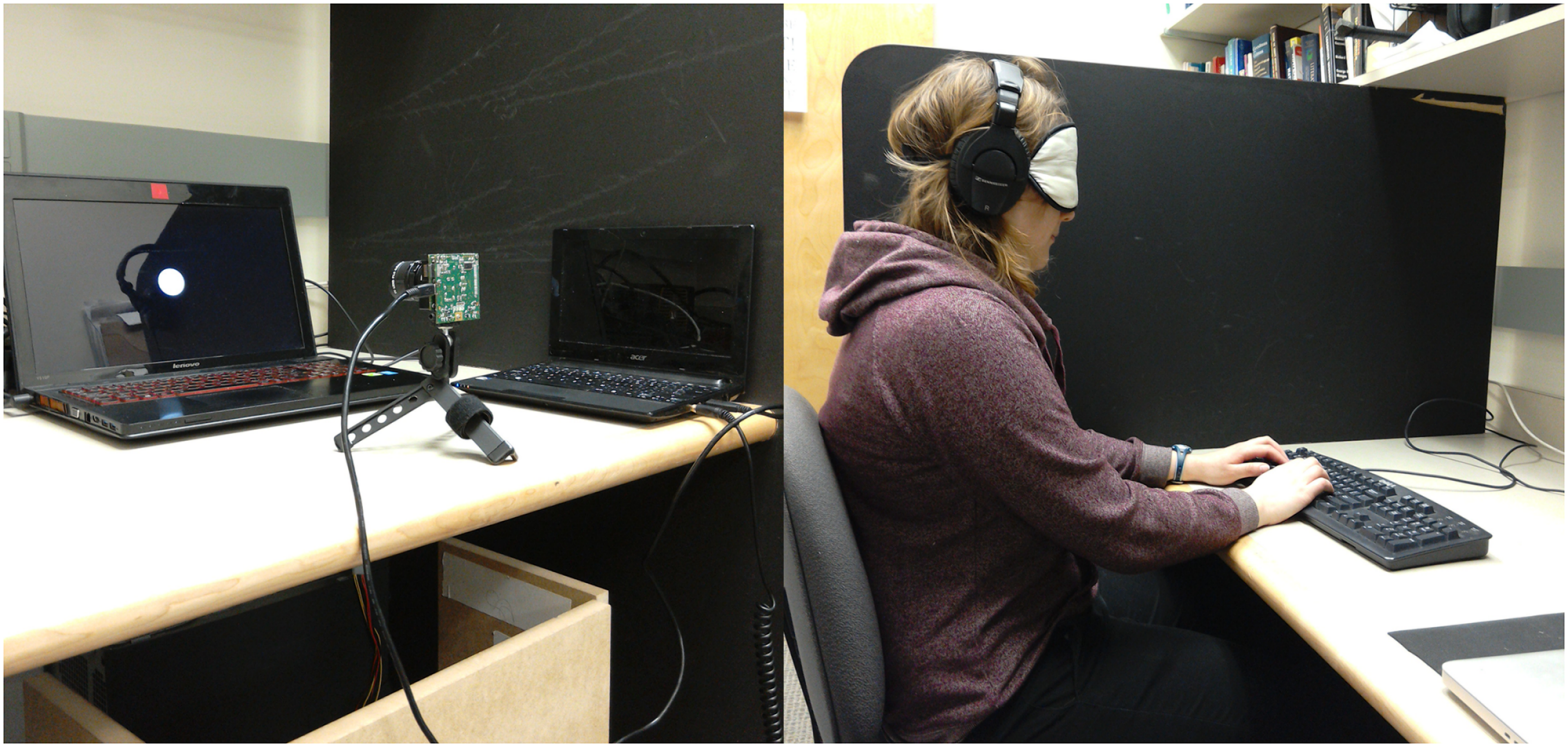
Experimental setup. Participants were blindfolded and positioned to the right of the black wooden separator to prevent any visual information from the laptop computer. The netbook was running the auditory augmented reality software and providing the headphone audio signal. The camera was positioned 42 cm away from the screen of the laptop to capture the entirety of the screen. The participant wore a blindfold to reduce the likelihood of visual distraction. The individual in this manuscript has given written informed consent (as outlined in PLOS consent form) to publish these case details.

The neuromorphic camera (DAVIS 240B, inilabs.com) was placed in front of a laptop computer (15.6” Lenovo Y510P, Intel i7-4700MQ) screen (1920x1080 60Hz) with custom MATLAB Psychophysics toolbox code [18] running to generate still and moving stimuli (Fig 1). The camera was placed 42 centimeters from the screen to ensure the entirety of the screen was captured. The camera was connected to a second laptop computer (Acer Aspire One D255E-1638, Intel Atom N570 1.66 GHz) to generate the spatial audio events of the scene. Clicks (i.e. a ‘1’ in the audio buffer) were generated with ITD and ILD modulation to create the percept of spatial azimuth. For each visual event generated by the neuromorphic camera, a matching auditory click was generated. Participants, wearing a pair of headphones (Sennheiser HD280, Sennheiser, USA), were seated next to the apparatus separated using a large wooden barrier to prevent events generated by the participant’s actions to be reflect on the stimulus laptop’s screen (and thus the DVS). The participant did not glean any visual information during the experiment.

### Algorithm of ITD and ILD

Custom software developed using C++ allowed for visual events generated by the neuromorphic sensor to be perceived in the auditory domain with the azimuthal spatial quality preserved. The interaural time difference is the difference of arrival time between the ears. To simulate this in a pair of headphones, one of the two channels of identical audio need to be shifted. The common audio sampling rate of 44.1 kHz was used, where shifting by 18 samples creates a 400 us shift in the sound (i.e. $\frac{18\;samples}{44100\;samples/second}=400\;us$). The ITD algorithm worked as follows: as a visual event occurs, the horizontal distance from the midline of the scene (95 pixels is the center, as there are 190 pixels across) was simply mapped to a value between -18 and +18. This value was used as a relative offset from the other channel to simulate the ITD cue. For example, if a visual event occurs in the far left of the scene (e.g. a midline offset distance of -95), the audio signal would be shifted by -18 samples in the audio channel’s buffer.

The ILD cue was generated by taking each event’s absolute horizontal distance from the scene’s midline, and attenuating the intensity of the audio. Each sample could be attenuated from a range of 0 to 95% (i.e. midline to far-right or far-left). For example, if a sound occurs in the extreme-far-right portion of the scene, the left channel’s audio would be attenuated by 95%.

### Onset detection and motion discrimination tasks

The study had two experiments: onset detection and motion discrimination.

For the onset detection phase, 100 trials were generated. There were an equal number of trials showing a stimulus (e.g. a white dot on a black screen) or a blank screen. Participants responded using an external keyboard to indicate whether a stimulus had been perceived in auditory space.

For the motion discrimination task, 500 trials were generated. Five different total displacements were used, ranging equally from 100 pixels to 400 pixels on the computer monitor (i.e. 100, 175, 250, 325, and 400 pixels or in visual degrees: 2.31, 4.05, 5.78, 7.52, and 9.25.). The displacement referred to the number of pixels the stimulus would move from the original starting point. The task was counterbalanced to include an equal number of displacements, equaling 100 total trials for each displacement, making up the total of 500 trials. Similarly, five different stimulus speeds were used, ranging from 5 to 20 pixels/frame (i.e. 5, 8.75, 12.5, 16.25, and 20 pixels/frame or in visual degrees/second: 7.20, 12.3, 17.4, 22.5, 27.6.). The task was run at a framerate of 60 Hz. Again, the task was counterbalanced to include an equal number of speeds, equally 100 total trials for each speed. These values were used to determine a threshold, should any exist, in either displacement or speed. Like the onset detection task, participants responded using an external keyboard to indicate whether a stimulus had moved either left or right.

Stimuli were pseudo randomly generated prior to beginning each task. Starting position was randomly chosen, and counterbalanced on the left and right. All participants had unique orders of stimulus presentation. Reaction time and hit rate was recorded for each trial. Reaction time was determined as the amount of time to respond after the stimulus first appeared on the screen.

### Timing of the algorithm execution time

An apparatus was built to calculate the time difference between the onset of the visual and auditory events. The time difference was consistently found to be in the range of 6–8 milliseconds, demonstrating that the algorithm was working in near-real-time. This test was performed to ensure there was not a large delay between visual and auditory onsets, meaning it can be used to react to stimuli in real-time.

### Analysis

All analysis was performed offline using SPSS Statistics 22.0 for Windows (SPSS Inc., Chicago, IL, USA).

### Hit rate (HR)

To determine performance on the task, each trial was evaluated and marked as either correct or incorrect when compared to the actual known movement of the stimulus for the trial. Hit rate was calculated as the number of correct responses divided by the number of incorrect responses.

### Reaction times (RT)

Reaction time analysis was performed to get a sense of the cognitive load required to complete the task. The more difficult the task, the more time it should take to complete. The reaction time was calculated as the amount of time between the visual onset and the key press.

## Results

### Onset detection HR

Hit rate was calculated as the average number of correct trials. Participants detected onsets with a 95.6% (sd: 9.1%) success rate.

### Motion discrimination HR

When collapsing across displacement and speed, participants discriminated the direction of motion with a 62.7% (sd: 3.81%) success rate.

### Displacement HR

**Fig 2:**
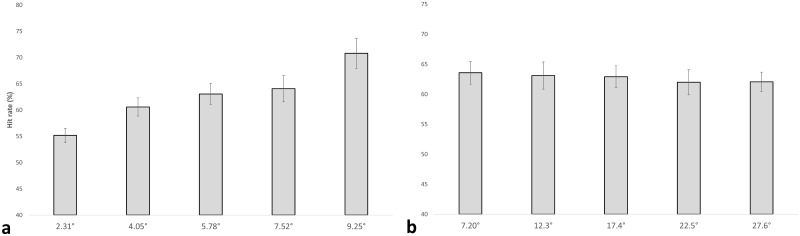
Hit rate during motion discrimination. (a) Bar graph showing the hit rate of each of the displacement trials. The horizontal axis contains the different displacements (in visual degrees) used. The respective matching displacements in pixels are 100, 175, 250, 325, and 400. The vertical axis is the average hit rate in percent. A significant effect of displacement was revealed, where more displacement was found to produce a higher hit rate. Standard error bars are shown. (b) Bar graph showing the hit rate for each of the displacement trials. The horizontal axis contains the different speeds (in visual degrees/second) used. The respective matching speeds in pixels/frame are 5, 8.75, 12.5, 16.25, and 20. The vertical axis is the average hit rate in percent. Standard error bars are shown.

Percent correct ranged from 55.247% (D100) to 70.699% (D400), with hit rate increasing with each increase in displacement (Fig 2a). Pairwise comparisons revealed significant differences between all measures of displacement, except for D175 & D250 and D250 & D325 (Table 1). A repeated-measures ANOVA revealed a significant effect of displacement (F(4, 72) = 16.945, p < 0.001).

**Table 1:** Paired-samples t-tests across all measures of displacement hit rate.

Pair	t statistic	p value
D100 & D175	-3.101	0.006164
D100 & D250	-3.806	0.001295
D100 & D325	-4.069	0.000721
D100 & D400	-5.754	0.000019
D175 & D250	-1.398	0.179058
D175 & D325	-2.282	0.034841
D175 & D400	-4.990	0.000095
D250 & D325	-.777	0.447102
D250 & D400	-4.251	0.000481
D325 & D400	-4.062	0.000732

All measures were found to be significant, except for D175 & D250 and D250 & D325.

### Speed HR

Most of the hit rates tended to be like one another (Fig 2b). Pairwise t-tests confirmed no significant difference in hit rate between any of the speed measures. A repeated-measures ANOVA revealed no significant effect of speed on hit rate.

### Onset detection RT

The average response time for the signal detection task was 2.02 seconds (sd: 2.00 seconds). Further investigation revealed two participants whose data influenced the mean with significantly longer reaction times (z-score > 2.2). Upon removal, the average response time was 1.38 seconds (sd: 0.71 seconds). When removing individual trials that exceeded a z-score of +-3 within each participant’s block, the average response time was found to be 0.94 seconds (sd: 0.43 seconds).

### Displacement RT

**Fig 3:**
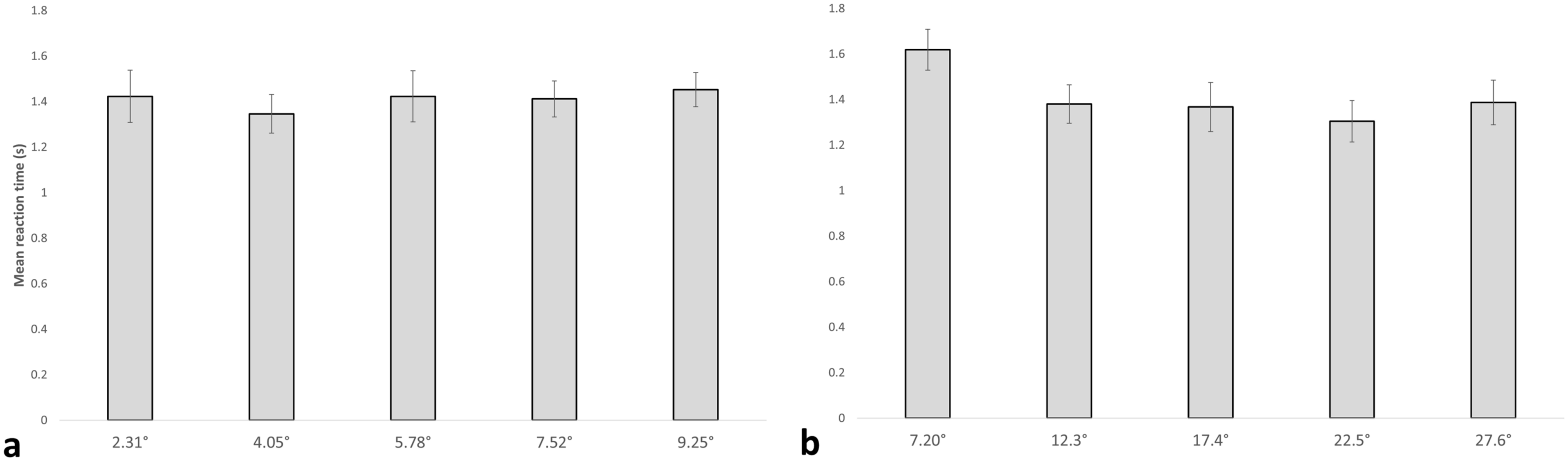
Mean reaction times during motion discrimination. (a) Bar graph showing the mean reaction time of each of the displacement trials. The horizontal axis contains the different displacements (in visual degrees) used. The respective matching displacements in pixels are 100, 175, 250, 325, and 400. The vertical axis contains the average reaction time in seconds. Standard error bars are shown. (b) Bar graph showing the reaction time for each of the speed trials. The horizontal axis contains the different speeds (in visual degrees/second) used. The respective matching speeds in pixels/frame are 5, 8.75, 12.5, 16.25, and 20. The vertical axis contains the average reaction time in seconds. Standard error bars are shown.

A repeated-measures ANOVA was performed, which revealed no significant effect of displacement (F(4,72) = 1.018, p = 0.361)) (Fig 3a), suggesting that reaction time does not change with displacement. Variance was not found to be equal within subjects for this analysis, and as such the Greenhouse-Geisser bounds were used to provide a more accurate F value.

### Speed RT

For speed, significant differences between S5 and S8.75 (t(18) = 9.356, p < 0.01), S5 and S12.5 (t(18) = 8.379, p < 0.01), S5 and S16.25 (t(18) = 6.047, p < 0.01), S5 and S20 (t(18) = 4.107, p < 0.01), S8.75 and S12.5 (t(18) = 2.988, p < 0.01) and S8.75 and S16.25 (t(18) = 2.206, p < 0.05) were revealed. A repeated-measures ANOVA revealed a significant effect of speed (F(4, 72) = 19.485, p < 0.001), with reaction times in S5 being significantly slower than the rest of the measures (Fig 3b).

### Guessing

Binary forced-choice tasks can produce results consistent with guessing. As such, it is necessary to perform a one-sample t-test to rule out the possibility that the participants are simply guessing. A one-sample t-test with a mean of 50 (percent) was performed on all data. All p-values were significant (all ps < 0.001), suggesting that any effect generated from the data was not due to guessing.

## Discussion

The present study investigated whether an auditory augmented-reality system could render visual events as salient auditory events and convey spatial information about the visual scene. The system converts purely-visual stimuli into auditory stimuli with preserved spatial characteristics. Participants were able, with no training and very little practice, to successfully detect a visual stimulus as well as determine the directionality of the stimulus in motion at a rate significantly better than chance. A neuromorphic camera allowed for the detection of visual stimuli with minimal power and processing requirements. Custom software to approximate the ITD and ILD cues allowed for the visual events encoded by the neuromorphic sensor to be perceived in the auditory domain.

A key functional advantage of the neuromorphic camera is its ability to extract the luminance dynamics of a scene without any further image processing. This means that algorithms dependent on brightness changes and moving edges can run on less powerful hardware than might otherwise be required with a conventional frame-based camera. Indeed, although the computer used to render the spatial audio signal was a low-power netbook, rendering visual AER events into spatial auditory events took between 6–8 milliseconds. In fact, the visual-to-audio rendering ran well on a Raspberry Pi 3 single-board computer, although limitations in that board’s native audio output made it unsuitable for the experiments described here.

In both tasks, reaction time (RT) was much slower than would be normally expected. An average reaction time across valid trials was 0.94 seconds (sd: 0.43 seconds) in the onset detection task and 1.34 seconds (sd: 0.70 seconds) in the motion discrimination task. As visual-to-audio latency was on the scale of a few milliseconds, these prolonged RTs were due to sensory and perceptual effects. One possible reason for this lengthy RT was the presence of occasional spurious noise events in the camera output, which appeared to users as occasional auditory events in the auditory scene with random locations and brief duration. In general, a high signal-to-noise ratio was achieved, with only occasional noise. However, some participants reported that it was difficult to filter out the extraneous noise events. We speculate that the prolonged RTs may have been due to the uncertainty involved with ignoring the noise stimuli, with additional time required to build evidence for the true onset of a visual event. An additional possible reason for the prolonged RT in the motion discrimination task is that the total stimulus presentation times varied randomly.

We found a main effect of displacement for motion discrimination. Essentially, larger displacement meant more time to build evidence for the direction of the motion for the trial, so this was expected. Speed, however, was not found to affect accuracy in any significant way. When collapsing across speed groups, a mean accuracy of 62.7% (sd: 0.69%) was found. This suggests that participants formed their judgments of motion direction using spatial displacement rather than velocity. One strategy could be to determine the start and end points of the task and simply figure out what direction was necessary to get from the start to the end.

It is likely that practice will improve performance, just like a new user of a prosthetic device (such as an arm or leg) may perform poorly at first, but will usually become proficient enough to replace the functionality of the lost limb. It is likely that even at very low displacements, a moving auditory stimulus would be unambiguous enough for the attention system to extract a cue about visual events.

The prototype device described here shows proof-of-concept and points toward a prosthetic device for patients with visual or attention orienting deficits. Currently, the system requires the user to wear a pair of headphones, which can interfere with the auditory system. As blind individuals are dependent on accurate auditory cues, this can pose a major problem. Passing through audio of the real world into the headphones would alleviate part of this problem, at the added expense of increased bulkiness of an added microphone. The most critical constraint relates to the rendering of real-world visual scenes with complex dynamics. When many visual events occur across the scene, the auditory augmented reality device becomes largely unusable. The auditory rendering fails to provide the cues needed to un-mix the complex auditory scene—a situation known as the Cocktail Party Problem. This might arise because several visual objects move simultaneously, but also occurs whenever the camera is panned across a stationary background. To handle complexity due to multiple visual events, future implementations will need to attach distinct auditory tags, possibly of varying pitch, as cues for the user to parse the acoustic scene. To handle egocentric motion of the camera, newer iterations of the system will require implementation of an event filter to remove background motion, probably at the cost of computational demands.

The DVS does include an inertial-measurement unit for this purpose and differentiating self-motion from moving targets is an active area of development [19, 20]. While the current implementation of the augmented auditory software does not include inputs from the inertial-measurement unit, it is a logical next step in development. When combined with visual grouping, the concept that the visual system tends to group related stimuli together (i.e. as uniform objects) [21, 22], the system described could potentially be used to solve extremely complex visual scenes and augment them to auditory space. The size of the camera proves to be a limiting factor, which can be attached to the body but is too large to be unnoticeable to the wearer. As neuromorphic technologies are currently in their infancy, newer, smaller, and faster technologies are likely to make neuromorphic vision sensors viable as wearable visual prosthetic devices.
